# A GENETIC ALGORITHM-BASED APPROACH TO OPTIMIZE THE CONSTRUCTION OF A FRAILTY INDEX

**DOI:** 10.1093/geroni/igz038.2530

**Published:** 2019-11-08

**Authors:** Alberto Zucchelli, Alessandra Marengoni, Debora Rizzuto, Amaia Calderon-Larranaga, Graziano Onder, Laura Fratiglioni, Davide Vetrano

**Affiliations:** 1 Department of Clinical and Experimental Sciences, University of Brescia, Brescia, Italy; 2 Aging Research Center, Department of Neurobiology, Care Sciences and Society, Karolinska Institutet and Stockholm University, Stockholm, Sweden; 3 Centro di Medicina dell’Invecchiamento, IRCCS Fondazione Policlinico “A. Gemelli” and Catholic University of Rome, Rome, Italy

## Abstract

The frailty index (FI) is a reliable prognostic indicator based on an individual clinical and functional deficits, which is strongly associated with poor outcomes. We hypothesize that an optimization algorithm may help to select the best candidate deficits to generate a highly-predictive FI. We aimed to optimize the predictive accuracy (area under the curve; AUC) of a FI employing a “genetic algorithm”, an iterative meta-heuristic that selects and recombines the most accurate FIs among randomly-generated ones. We used data from 3363 individuals aged 60+ enrolled in the Swedish National Study on Aging and Care in Kungsholmen (SNAC-K). To avoid overfitting, the algorithm was run on a randomly-chosen subsample (70%) of 10 imputed datasets. About 825,000 FIs were built, evaluated, and recombined. The best genetic algorithm-based FI (ga-FI) was compared in terms of 3- and 6-year mortality prediction with a clinically-generated FI (c-FI) in the remaining 30% of the data. Ga-FI showed better AUCs in comparison to the c-FI, overall and in all age and sex subsamples. Several sensitivity analyses were carried out. The major AUC improvement was seen among participants aged <75 [3-year mortality AUC: 0.83 vs 0.63; p<0.001]; 6-year mortality AUC: 0.76 vs 0.63; p<0.001], while smaller differences were seen among participants aged ≥75 [3-year mortality AUC: 0.86 vs 0.84; p=0.216; 6-year mortality AUC: 0.84 vs 0.81, p=0.017]. The genetic algorithm is a feasible method to optimize the construction of a highly performant FI that might be used to assess health comprehensively both in clinical and research settings.

